# Fisetin Delays Postovulatory Oocyte Aging by Regulating Oxidative Stress and Mitochondrial Function through *Sirt1* Pathway

**DOI:** 10.3390/molecules28145533

**Published:** 2023-07-20

**Authors:** Xupeng Xing, Yalin Liang, Yanan Li, Yaolu Zhao, Yuxing Zhang, Zheng Li, Zicong Li, Zhenfang Wu

**Affiliations:** 1 National Engineering Research Center for Breeding Swine Industry, South China Agricultural University, Guangzhou 510642, China; 2 Gene Bank of GuangDong Local Livestock and Poultry, College of Animal Science, South China Agricultural University, Guangzhou 510642, China; 3 State Key Laboratory of Livestock and Poultry Breeding, South China Agricultural University, Guangzhou 510642, China

**Keywords:** fisetin, postovulatory oocyte aging, oocyte quality, embryo development, *Sirt1*

## Abstract

The quality of oocytes determines the development potential of an embryo and is dependent on their timely fertilization after ovulation. Postovulatory oocyte aging is an inevitable factor during some assisted reproduction technology procedures, which results in poor fertilization rates and impairs embryo development. We found that fisetin, a bioactive flavonol contained in fruits and vegetables, delayed postovulatory oocyte aging in mice. Fisetin improved the development of aged oocytes after fertilization and inhibited the *Sirt1* reduction in aged oocytes. Fisetin increased the GSH level and *Sod2* transcription level to inhibit ROS accumulation in aged oocytes. Meanwhile, fisetin attenuated aging-induced spindle abnormalities, mitochondrial dysfunction, and apoptosis. At the molecular level, fisetin decreased aging-induced aberrant expression of H3K9me3. In addition, fisetin increased the expression levels of the mitochondrial transcription factor *Tfam* and the mitochondrial genes *Co2* and *Atp8* by upregulating *Sirt1* in aged oocytes. Finally, inhibition of *Sirt1* reversed the anti-aging effects of fisetin. Taken together, fisetin delayed postovulatory oocyte aging by upregulating *Sirt1*.

## 1. Introduction

Metaphase I (MI) oocytes undergo maturation and are arrested in the metaphase II (MII) stage until fertilization. If MII oocytes are not fertilized at the proper time after ovulation, they lose their developmental competence, a phenomenon known as postovulatory oocyte aging. Despite the occurrence of postovulatory aging in both in vivo and in vitro settings, it has consistently been linked to negative outcomes such as decreased fertilization rates, suboptimal embryo quality, errors during the post-implantation stage, and various abnormalities in the offspring [1]. Postovulatory aged oocytes exhibit several detrimental effects, including partial cortical granule exocytosis, impaired mitochondrial function, zona hardening, abnormalities in the spindle structure, and alterations in epigenetic patterns [1]. In mice, the quality of oocytes rapidly deteriorates 6 h after ovulation if they are not fertilized, with them subsequently being lost after 12 h [2]. For human oocytes, the time window for fertilization is 4–12 h after ovulation [3]. Therefore, timely fertilization of oocytes is crucial to ensure the development of embryos. Assisted reproduction technology (ART) has been widely used over the last few decades for treating infertility. However, ART procedures inevitably result in aging after ovulation or retrieval. Intracytoplasmic sperm injection (ICSI) is used to fertilize oocytes in case of failed in vitro fertilization (IVF) [4]. Although oocytes fertilized via ICSI can develop into a viable embryo, the procedure takes 6–12 h and hence induces postovulatory oocyte aging [5]. Not surprisingly, aging-induced defects such as oxidative stress, mitochondrial dysfunction, and chromosomal abnormalities have been detected in oocytes during ART procedures, which can result in a poor oocyte quality, lower fertilization rates, embryo development aberrancy, and even unhealthy offspring [6,7,8]. Therefore, it is imperative to develop strategies to improve the outcomes of IVF. 

Fisetin (3,3′,4′,7-tetrahydroxyflavone), a bioactive flavonol, is abundant in strawberries, apples, onions, and cucumbers [9,10] and has distinct antioxidant properties. Flavonols, a type of flavonoid compound, possess a ketone group and serve as fundamental components of proanthocyanins [11]. These compounds are abundantly found in various fruits and vegetables. Among the well-studied flavonols are kaempferol, quercetin, myricetin, and fisetin [11]. In addition to fruits and vegetables, tea and red wine also serve as sources of flavonols [11,12]. The function of flavonols has been linked to numerous health benefits, including the effects of their antioxidant properties and a reduced risk of vascular diseases [11]. Fisetin has been proven to be chemopreventive, neuroprotective, anti-inflammatory, anticancer, and anti-apoptotic [13,14]. It can inhibit ROS production by increasing the expression of antioxidant enzymes while decreasing that of oxidant enzymes [15]. Recent studies have shown that fisetin can extend the lifespan of animals [16]. Aging-induced phenotypes in oocytes include oxidative stress, DNA damage, mitochondrial dysfunction, apoptosis, spindle abnormalities, and abnormal morphology [17,18]. Oxidative stress triggers mitochondrial dysfunction, impairs calcium homoeostasis, and induces DNA damage. Dysfunctional mitochondria in aged oocytes are characterized by an abnormal distribution, a low mitochondrial membrane potential, and less ATP production [17]. The mitochondrial DNA (mtDNA)-encoded genes *Co2* and *Atp8* are components of the ATP-generating oxidative respiratory chain, and mutations in mtDNA can increase the production of reactive oxygen species (ROS), which eventually results in a vicious cycle of oxidative stress and mitochondrial dysfunction [19,20]. However, a possible therapeutic effect of fisetin on oocyte aging and mitochondrial function has not been investigated thus far.

Sirtuins (SIRTs) are NAD^+^-dependent histone deacetylases that regulate cell survival, apoptosis, DNA repair, and metabolism, among others. *Sirt1* plays crucial roles in extending lifespan [21] and also regulates postovulatory oocyte aging by controlling ROS accumulation, spindle abnormalities, apoptosis, and mitochondrial dysfunction [17,22]. It can regulate mitochondrial metabolism and mtDNA transcription via the deacetylation of peroxisome proliferator-activated receptor gamma coactivator-1α (PGC-1α) [23]. The levels of acetylated and methylated histones such as H4K8ac, H4K12ac, and H3K9me3 undergo significant changes during postovulatory oocyte aging [18,24]. Thus, *Sirt1* may exert protective functions by regulating histone acetylation in oocytes. Fisetin can inhibit IL-1β-induced inflammatory responses, oxidative stress, and hepatic steatosis in humans and mice by increasing SIRT1 activity [25,26,27]. However, it is still unknown whether fisetin affects *Sirt1* in oocytes.

In the present study, we found that fisetin promoted the development of aged oocytes after fertilization, reversed the non-epigenetic and epigenetic changes during postovulatory oocyte aging, and restored mtDNA transcription by increasing SIRT1 activity.

## 2. Results

### 2.1. Fisetin Promoted the Development Potential of Aged Oocytes after Fertilization

**Figure 1:**
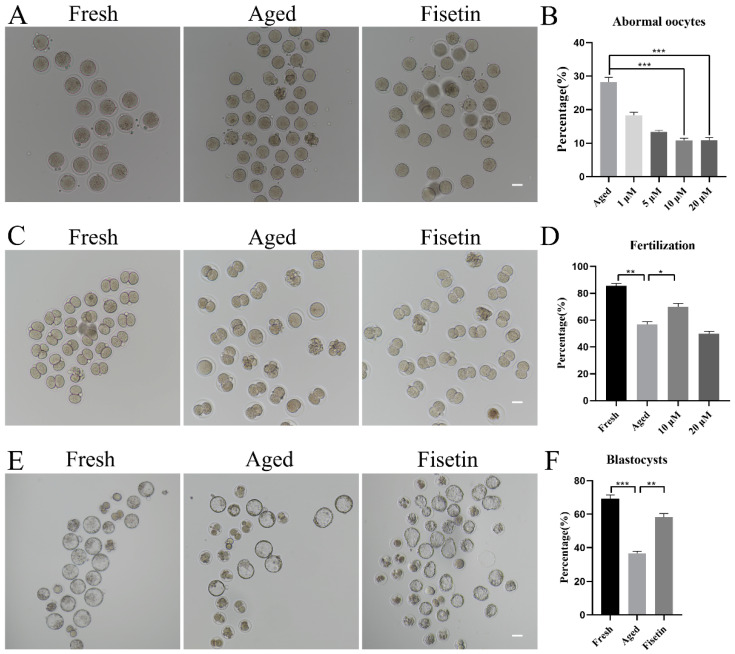
Effects of fisetin on oocyte morphologies, fertilization, and blastocyst formation during postovulatory oocyte aging. (**A**) Representative images of the fresh, aged, and 10 µM fisetin-treated oocytes. Scale bar is 50 µm. (**B**) the percentages of abnormal oocytes in the indicated groups (n = 90 for each group). (**C**,**D**) Representative images of 2-cell and the ratio of fertilization in the fresh, aged, and 10 µM fisetin-treated oocytes (n = 90 for each group). Scale bar is 50 µm. (**E**,**F**) Representative images of blastocysts and the rate of blastocyst formation after 120 h in the fresh, aged, and 10 µM fisetin-treated oocytes (n = 90 for each group). Scale bar is 100 µm. * *p* < 0.05, ** *p* < 0.01, *** *p* < 0.001.

To determine the optimal dose of fisetin for delaying postovulatory oocyte aging, we treated MII oocytes with different doses of fisetin (1, 5, 10, and 20 μM) in vitro. The proportion of morphologically aberrant oocytes, including fragmental cells, decreased after 12 h of exposure to fisetin, and the reduction was most significant with 10 μM and 20 μM of the drug (Figure 1A,B). The rates of fertilization in the fresh, aged, and 10 μM fisetin-treated oocytes were 85.53%, 56.80%, and 69.98%, respectively (Figure 1C,D). Surprisingly, 20 μM fisetin led to a significant decrease in the fertilization rate compared with the lower dose of 10 µM (49.89 ± 3.25% vs. 69.98 ± 2.57%, *p* < 0.05; Figure 1D). The effect of fisetin on the development potential of oocytes was determined by analyzing the rate of blastocyst formation at 120 h after fertilization. The percentage of blastocysts formed by oocytes treated with 10 μM fisetin was significantly higher in comparison with the untreated aged oocytes (58.16 ± 3.22% vs. 36.62 ± 1.78%, *p* < 0.05; Figure 1E,F). Taken together, 10 μM fisetin not only reduced the rate of aging-induced abnormalities in oocytes but also increased their development potential after fertilization. Therefore, this dosage was used in subsequent experiments.

### 2.2. Fisetin Elevated Sirt1 Expression during Oocyte Aging

**Figure 2:**
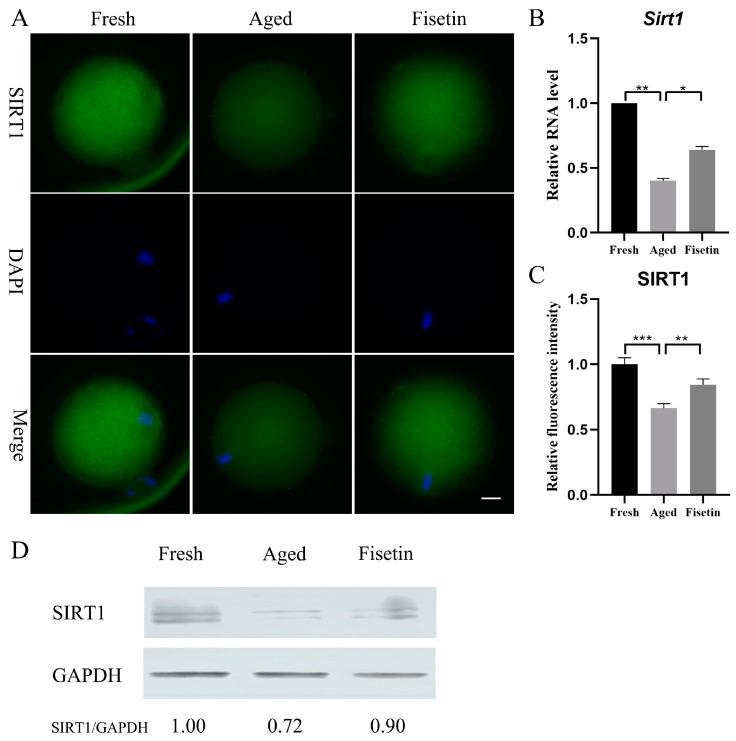
Fisetin affected *Sirt1* expression during postovulatory oocyte aging. (**A**) Representative images of SIRT1 in the fresh, aged, and fisetin groups. Scale bar is 20 µm. (**B**) The mRNA levels of *Sirt1* in the three groups. (**C**) The staining intensity of SIRT1 measured in the three groups (n = 20 for each group). (**D**) Representative Western blot results of SIRT1 in the three groups (n = 200 for each group). * *p* < 0.05, ** *p* < 0.01, *** *p* < 0.001.

*Sirt1* plays a crucial role during postovulatory oocyte aging [17,18] and has been shown to be upregulated by fisetin [25,26]. However, it is unknown whether fisetin can affect *Sirt1* expression in oocytes. Here, the mRNA level and the protein level of SIRT1 significantly decreased in the aged groups, and these were significantly restored in the fisetin groups (*p* < 0.05; Figure 2A–C). Then, the results of the Western blot analysis also demonstrated that the intensity of the SIRT1 band decreased in the aged groups. However, upon supplementation with fisetin, there was a noticeable increase in the intensity of the SIRT1 band (Figure 2D). Collectively, fisetin prevented aging-induced SIRT1 abatement during postovulatory oocyte aging. 

### 2.3. Fisetin Inhibited Oxidative Stress by Regulating SIRT1 in Aged Oocytes

**Figure 3:**
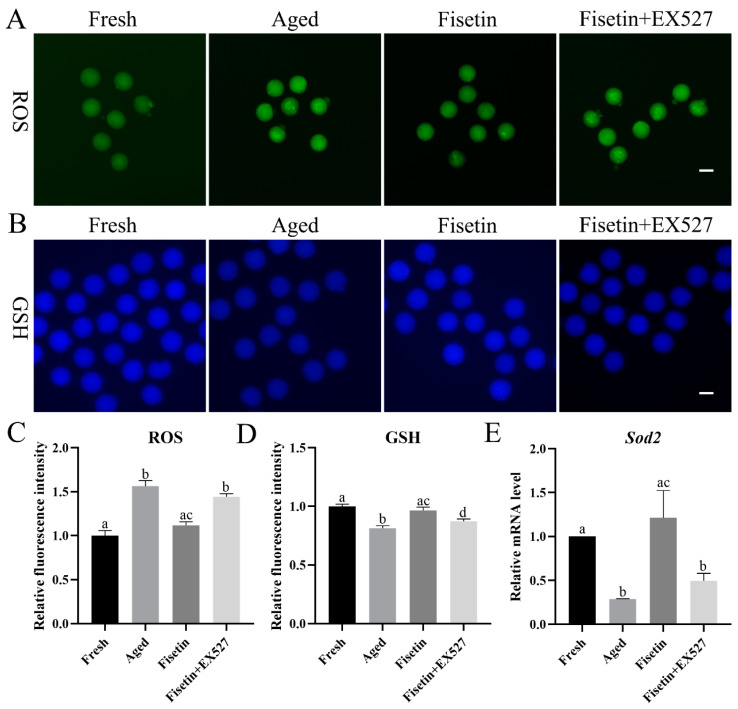
Fisetin regulated oxidative stress by controlling SIRT1 activity. (**A**,**C**) Representative images of ROS and the relative ROS intensities analyzed in the fresh, aged, fisetin, and fisetin + EX527 groups (n = 20 for each group). Scale bar is 60 µm. (**B**,**D**) Representative images of GSH and the relative GSH intensities analyzed in the four indicated groups (n = 20 for each group). Scale bar is 60 µm. (**E**) Analysis of the relative mRNA of Sod1 in the four indicated groups. The different superscripts a, b, c, and d represent significant differences at *p* < 0.05.

Oxidative stress is the main course of postovulatory oocyte aging [1]. We found that ROS accumulation increased significantly in the aged oocytes and was reduced by fisetin (*p* < 0.05; Figure 3A,C). To determine whether fisetin reversed the aging-induced changes in oocytes by upregulating *Sirt1*, we treated the cells with the SIRT1 inhibitor EX527 (IC50, 123 nM) during postovulatory aging. Since both 100 nM and 200 nM EX527 inhibited the activity of SIRT1 in oocytes and pancreatic beta cells [18,28], the concentration of 200 nM was used in the present study. After inhibiting SIRT1 with EX527, ROS accumulation increased significantly compared with that in the fisetin-treated groups (*p* < 0.05; Figure 3A,C).

Glutathione (GSH) has antioxidant ability. The concentrations of GSH significantly decreased in the aged oocytes. The fisetin treatment significantly increased the fluorescence intensity of GSH, and this was reversed by inhibiting SIRT1 with EX527 (*p* < 0.05; Figure 3B,D). 

SIRT1 inhibits ROS accumulation through the MnSOD (*Sod2*) pathway [29]. The transcript level of *Sod2* was detected during postovulatory oocyte aging. The mRNA level of *Sod2* was downregulated in the aged oocytes, which was rescued with the fisetin treatment and significantly reversed by the inhibition of SIRT1 (*p* < 0.05; Figure 3E). Taken together, fisetin attenuated oxidative stress with increasing GSH and *Sod2* levels by increasing SIRT1 activity in the aged oocytes.

### 2.4. Fisetin Attenuated Abnormal Spindle Formation, γH2A.X Reduction, and Apoptosis in Aged Oocytes

**Figure 4:**
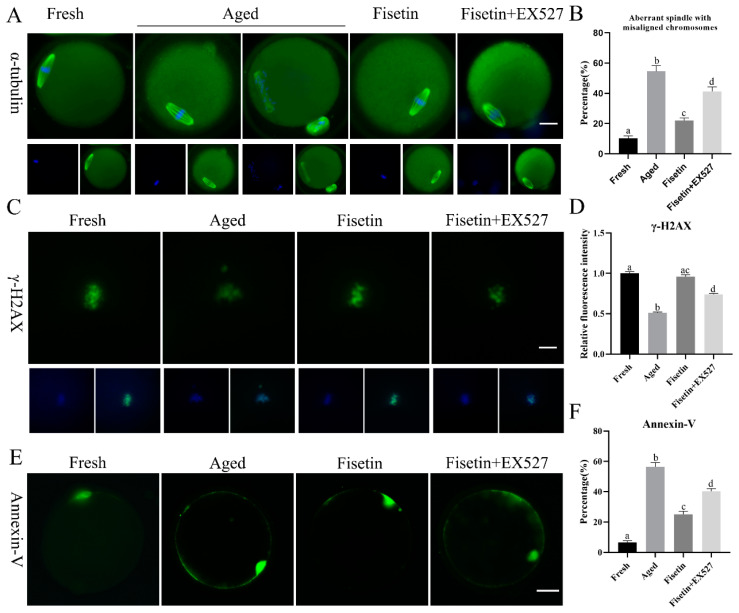
Fisetin affected spindle morphology, γH2A.X foci, and apoptosis during postovulatory oocyte aging. (**A**,**B**) Representative images of spindles with DNA and the rate of abnormal spindles with misaligned chromosomes measured in the four groups (n = 60 for each group). Scale bar is 25 µm. (**C**,**D**) Representative images of γH2A.X and the relative intensity of γH2A.X analyzed in the four groups (n = 20 for each group). Scale bar is 10 µm. (**E**,**F**) Representative images of Annexin-V and the ratio of Annexin-V signals analyzed in the four groups (n = 60 for each group). Scale bar is 25 µm. The different superscripts a, b, c, and d represent significant differences at *p* < 0.05.

Spindle defects, DNA damage, and apoptosis are the pathological manifestations of postovulatory oocyte aging. The typical barrel-shaped spindle with aligned chromosomes ensures that the chromosomes are evenly divided into two sister cells after fertilization [30]. As shown in Figure 4A, the typical spindle with well-aligned chromosomes was disrupted in the aged oocytes, and the rate of abnormal spindle formation was significantly increased after 12 h of in vitro aging compared to that in the fresh oocytes (54.53 ± 5.47% vs. 10.37 ± 2.10%, *p* < 0.05; Figure 4B). However, fisetin significantly restored normal spindle formation in the aged oocytes, in contrast to their untreated counterparts (21.93 ± 2.43% vs. 54.53 ± 5.47%, *p* < 0.05; Figure 4B). Oocytes co-treated with fisetin and EX527 showed significantly higher rates of abnormal spindle formation in comparison with their fisetin-treated counterparts (41.20 ± 4.31% vs. 21.93 ± 2.43%, *p* < 0.05; Figure 4B).

Foci of the phosphorylation of histone H2A.X at serine 139 (γH2A.X) indicate the initiation of DNA repair [31]. γH2A.X foci are molecular markers of DNA damage [32]. The immunostaining result showed that γH2A.X accumulated on the chromosomes in the fresh oocytes (Figure 4C), and the intensity of the signals significantly decreased in the aged oocytes (*p* < 0.05; Figure 4C,D). The fisetin treatment significantly increased the intensity of the signals, and this was reversed by inhibiting SIRT1 with EX527 (*p* < 0.05; Figure 4C,D).

The apoptosis rates of the oocytes were analyzed via Annexin-V staining, and the green fluorescence on the outer membrane of the oocytes was recorded (Figure 4E). Apoptosis was significantly higher in the aged oocytes compared to the fresh oocytes (56.46 ± 3.90% vs. 6.50 ± 1.55%, *p* < 0.05; Figure 4F). The fisetin treatment of the aged oocytes markedly decreased the percentage of apoptotic cells (25.10 ± 2.79% vs. 56.46 ± 3.90%, *p* < 0.05, Figure 4F), whereas the co-treatment with fisetin and EX527 significantly increased the rate of apoptosis in the oocytes compared with the fisetin-treated groups (56.46 ± 3.90% vs. 40.33 ± 2.46%, *p* < 0.05, Figure 4F). Taken together, fisetin can partially alleviate abnormal spindle assembly, γH2AX accumulation, and apoptosis by regulating SIRT1 during postovulatory oocyte aging.

### 2.5. Fisetin Attenuated the Aging-Induced Dysfunction of Mitochondria

**Figure 5:**
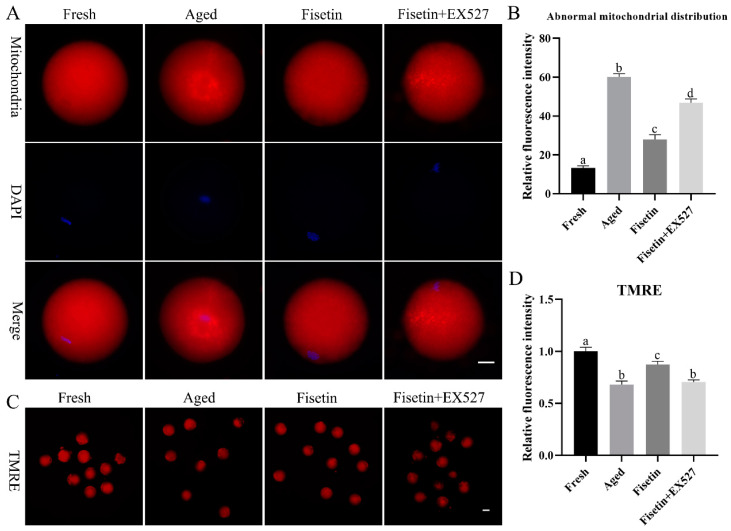
Fisetin restored mitochondrial function during postovulatory oocyte aging. (**A**,**B**) Representative images of the mitochondrial distribution and percentage of fresh, aged, fisetin-treated, and fisetin + EX527-treated oocytes with an abnormal mitochondrial distribution (n = 60 for each group). Scale bar is 25 µm. (**C**,**D**) Representative images showing TMRE-stained oocytes and the relative intensity of TMRE signals in the indicated groups (n = 20 for each group). Scale bar is 60 µm. The different superscripts a, b, c, and d represent significant differences at *p* < 0.05.

Mitochondrial function is an indicator of the quality of MII oocytes [7]. Therefore, we also evaluated the effects of fisetin on the mitochondrial distribution and mitochondrial membrane potential during postovulatory oocyte aging. As shown in Figure 5A, mitochondria were distributed evenly in the cytoplasm of the fresh oocytes but gathered into clusters after aging for 12 h. The rate of abnormally distributed mitochondria was significantly raised in the aged oocytes (60.17 ± 2.42% vs. 13.25 ± 1.61%, *p* < 0.05; Figure 5B) compared with the fresh groups, and this was significantly recovered by the fisetin treatment (27.97 ± 3.41% vs. 60.17 ± 2.42%, *p* < 0.05; Figure 5B). However, it was significantly higher with a skewed mitochondrial distribution in the fisetin + EX527-co-treated groups compared with the fisetin-treated groups (27.97 ± 3.41% vs. 46.74 ± 2.95%, *p* < 0.05; Figure 5B).

TMRE (tetramethylrhodamine) is an orange-red probe that accumulates in intact mitochondria as opposed to depolarized mitochondria [33]. The fresh oocytes with functional mitochondria emitted an intense red fluorescence, and the intensity was significantly lower in the aged oocytes (*p* < 0.05; Figure 5C,D), indicating that the mitochondrial membrane potential (MMP) decreased during oocyte aging. The fisetin treatment upregulated the MMP in the aged oocytes (*p* < 0.05; Figure 5C,D). However, the rescued MMP in the fisetin-treated oocytes was disrupted by the EX527 treatment (*p* < 0.05; Figure 5C,D). Altogether, fisetin prevented mitochondrial dysfunction through the SIRT1 pathway during postovulatory aging.

### 2.6. Fisetin Altered the Expression of Mitochondrial Genes in Aged Oocytes

**Figure 6:**
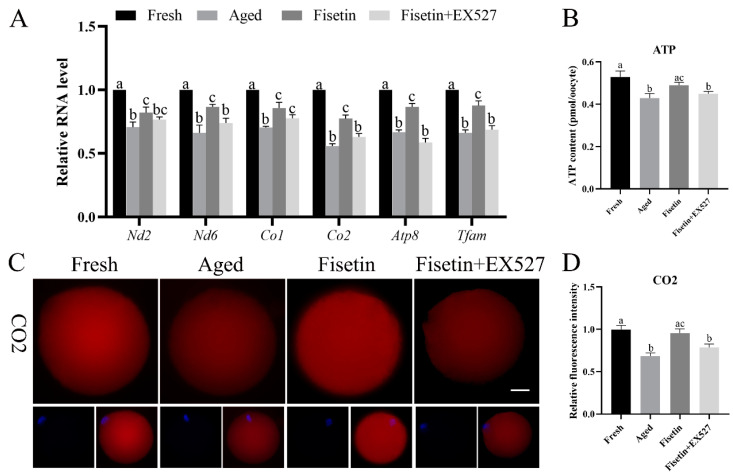

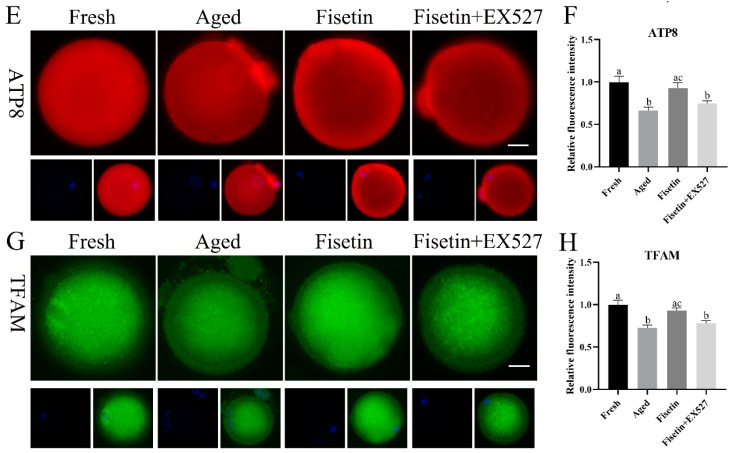
Fisetin altered mitochondrial gene expression by upregulating SIRT1 during postovulatory oocyte aging. (**A**) The levels of *Nd2*, *Nd6*, *Co1*, *Co2*, *Atp8*, and *Tfam* mRNAs in the fresh, aged, fisetin-treated, and fisetin + EX527-treated oocytes. (**B**) The ATP content in single oocytes from the indicated groups (n = 60 for each group). (**C**) Representative images showing in situ expression of Co2 and (**D**) the relative intensity in the four groups (n = 20 for each group). Scale bar is 25 µm. (**E**) Representative images showing in situ ATP8 expression and (**F**) the relative intensity in the four groups (n = 20 for each group). (**G**) Representative images showing in situ TFAM expression and (**H**) the relative intensity in the four groups (n = 20 for each group). The different superscripts a, b, and c represent significant differences at *p* < 0.05.

*Sirt1* regulates mitochondrial function via the PGC-1α/NRF1/TFAM signaling pathway [23]. Given the low mitochondrial membrane potential and ATP content observed during postovulatory oocyte aging, we therefore analyzed the expression of TFAM and mitochondrial respiratory chain genes in the aged oocytes. The qPCR results showed that the *Nd2*, *Nd6*, *Co1*, *Co2*, *Atp8*, and *Tfam* mRNA levels were significantly lower in the aged oocytes compared with the fresh oocytes (*p* < 0.05; Figure 6A). The fisetin treatment significantly upregulated the transcription level of *Tfam*, *Co2*, and *Atp8*, and this effect was reversed by inhibiting SIRT1 with EX-527 (*p* < 0.05; Figure 6A). In addition, the immunostaining results also showed a lower in situ expression of TFAM, Co2, and ATP8 in the aged oocytes, which increased significantly after the treatment with fisetin but was downregulated following SIRT1 inhibition (*p* < 0.05; Figure 6C–H). Consistent with these results, the ATP content decreased in the aged oocytes and was reversed by the fisetin treatment (0.49 ± 0.014 pmol vs. 0.43 ± 0.014 pmol, *p* < 0.05; Figure 6B). However, pharmacological inhibition of SIRT1 activity reversed the fisetin-induced increase in ATP production (0.49 ± 0.014 pmol vs. 0.45 ± 0.011 pmol, *p* < 0.05; Figure 6B). Taken together, fisetin may improve mitochondrial function in aged oocytes by altering the expression of mitochondrial genes through the regulation of SIRT1.

### 2.7. Fisetin Attenuated the Aberrant Intensity of H3K9me3

**Figure 7:**
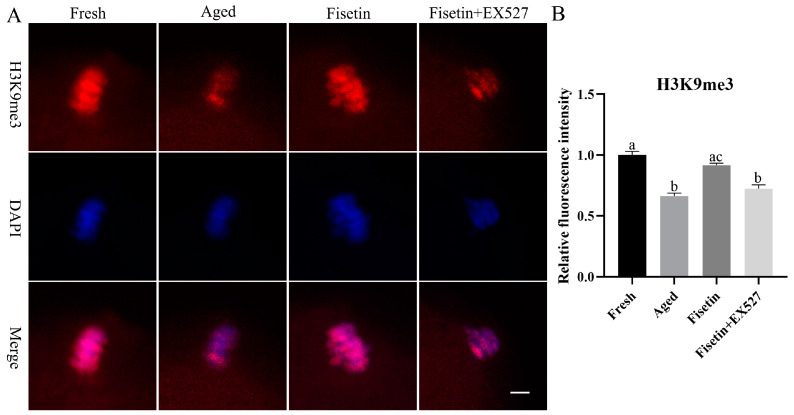
Fisetin affected the intensity of H3K9me3 during postovulatory oocyte aging. (**A**) Representative images of H3K9me3 signals in the fresh, aged, fisetin-treated, and fisetin + EX527-co-treated oocytes. Scale bar is 5 µm. (**B**) The relative intensity of H3K9me3 signals in the four indicated groups (n = 20 for each group). The different superscripts a, b, and c represent significant differences at *p* < 0.05.

SIRT1 regulates the tri-methylation of H3K9 (H3K9me3) in colitis and deacetylates the acetylated forms of H3K56 and H3K4 in cancer cells [34,35,36]. As shown in Figure 7A, H3K9me3 accumulated in the chromosomes of oocytes in the MII stage. The intensity of H3K9me3 significantly decreased in the aged oocytes compared to the fresh oocytes (*p* < 0.05; Figure 7A,B). The signals of H3K9me3 increased significantly in the fisetin-treated cells; however, they significantly decreased in the fisetin + EX527-co-treated oocytes (*p* < 0.05; Figure 7B). On the other hand, H3K56ac and H3K4ac were localized in the cytoplasm of the oocytes, and there were no signals on the chromosomes of either the fresh or aged oocytes (Figures S1 and S2 and Table S1). Taken together, fisetin recovered H3K9me3 in the aged oocytes.

## 3. Discussion

Postovulatory oocyte aging is an inevitable phenomenon during some ART procedures, which leads to a poor oocyte quality and poor embryo development [3]. Fisetin, a bioactive flavonol present in various fruits and vegetables, prevented postovulatory oocyte aging in mice. It not only maintained the quality of the oocytes during oocyte aging but also promoted the development of embryos after fertilization. Furthermore, aging-induced defects in the oocytes, such as excessive ROS production, apoptosis, spindle abnormalities, mitochondrial dysfunction, and epigenetic changes, were rescued by the fisetin treatment. Finally, fisetin increased the expression of *Sirt1*, a histone deacetylase, which was downregulated in the aged oocytes. Pharmacological inhibition of SIRT1 reversed the anti-aging effects of fisetin. Thus, fisetin might delay postovulatory oocyte aging during ART procedures.

Parts of the oocytes were fragmented after 12 or 24 h of induced aging, regarded as the loss of development potential [3,17]. Fisetin prevented aging-induced morphological abnormalities in the oocytes, which coincided with improved rates of fertilization and blastocyst formation. Interestingly, while both 10 μM and 20 μM fisetin prevented morphological defects during oocyte aging, the higher dose partially decreased the rates of fertilization. This may be related to fisetin-induced autophagic cell death or its effects on the cell cycle and invasion [37]. Therefore, the optimal concentration of fisetin may depend on the species.

Oxidative stress is the key initiator of postovulatory oocyte aging and triggers mitochondrial damage and apoptosis [1]. In agreement with previous studies, the aged oocytes showed increased ROS accumulation and apoptosis, which were significantly alleviated by fisetin [38,39,40]. In addition, *Sirt1* expression was low in the aged oocytes and upregulated by fisetin, which is consistent with a previous study [22]. Additionally, fisetin can efficiently increase the expression of *Sirt1* during oocyte aging. Since SIRT1 can promote ROS scavenging via the MnSOD pathway [18], fisetin may exert antioxidant effects in aged oocytes by inducing *Sirt1* expression. The mitotic spindle and chromosomal arrangement were also disrupted in the postovulatory aged oocytes [24,41]. Fisetin prevented spindle abnormalities in the oocytes, most likely by promoting SIRT1-mediated deacetylation of acetylated α-tubulin [17,22,42]. Thus, fisetin rescued the oocytes from aging-induced pathological changes by regulating *Sirt1* expression.

Double-strand breaks (DSBs) occur in cells, which are induced by aging, radiation, and genotoxic compounds [43]. γH2A.X recruits DNA damage response proteins to the site of DSBs [43]. γH2A.X foci, as makers of DNA damage, are the first indication of DNA repair [32]. SIRT1 has a critical role in modulating γH2A.X foci formation, indicated by the decrease in γH2A.X foci in cells with SIRT1 deficiency [44]. Consistent with this, the present results showed that γH2A.X on chromosomes was impaired in the aged oocytes, and increasing or inhibiting SIRT1 activity led to changes in the γH2A.X signals. In addition, γH2A.X foci formation appearing in the G2/M stage does not indicate the occurrence of DNA damage in mitotic cells [45,46]. The signals of γH2A.X appear on chromosomes in the MI stage during in vitro oocyte maturation [47]. A bright spot of γH2A.X has even been found in MII oocytes, which was not affected by the toxicity treatment or the vitrification process [48]. Above all, the signals of γH2A.X on chromosomes may not result from DNA damage in oocytes. However, it is attractive and meaningful to investigate the function of γH2A.X in the absence of DNA damage in oocytes.

Mitochondrial function is critical for the development potential of oocytes, and several studies have shown that mitochondria are dysfunctional in aged oocytes [49]. Fisetin restored the mitochondrial membrane potential in the aged oocytes, which can be attributed to its antioxidant effects and the increased expression of *Sirt1*. Studies show that *Sirt1* can regulate mitochondrial biogenesis by activating transcriptional factors such as *PGC-1α* and *HIF-1α* [23]. In addition, *Sirt1* and *PGC-1α* are also co-localized in mitochondria, both regulating mtDNA replication and transcription [50]. We found that fisetin regulated the expression of mitochondrial genes during postovulatory oocyte aging. Specifically, fisetin restored the expression levels of *Tfam*, *Co2*, and *Atp8* by increasing SIRT1 activity. *Co2* and *Atp8* are components of the oxidative respiratory chain, and Atp8 is responsible for the generation of ATP [19]. Consistent with this, fisetin also increased the ATP content in the aged oocytes. Considering that glycolysis, the mitochondrial respiratory chain, and the tricarboxylic acid (TCA) cycle form the fundamental pathways of mitochondria [51], epigenetic processes depend on the TCA cycle and the energy supplied by the mitochondrial respiratory chain, which are crucial for oocyte competence and embryo viability [52]. It is worth noting that energy metabolism is impaired in aged oocytes, and, therefore, further research is needed to investigate the status of the TCA cycle in aged oocytes. To summarize, the loss of MMP and ATP production in aged oocytes is likely due to the downregulation of Sirt1, which has a negative impact on mitochondrial biogenesis and gene expression. 

We have shown, for the first time, that fisetin can regulate the methylation of histone lysine. Postovulatory aging of oocytes is accompanied by a decrease in H3K9me3 levels [24], which was observed in our study. SIRT1 regulates the H3K9me3 level by deacetylating SUV39h1, which is the methyltransferase of H3K9me3 [42,53]. Although H3K4ac and H3K56ac are also deacetylated by SIRT1, we did not detect these histones on the oocyte chromosomes. It is possible that the histone acetylation was cleared from the chromosomes due to transcriptional silencing in the oocytes.

Oocytes from aged mice and postovulatory aged oocytes from adult mice share common defects such as oxidative stress, mitochondrial dysfunction, morphological abnormalities, and loss of developmental competence [1,54]. Resveratrol, as a natural polyphenolic present in grapes and wine, can extend the lifespan of many organisms, rescue maternal aged oocytes, and delay postovulatory oocyte aging in mice by activating *Sirt1* [55,56]. In the present study, fisetin also delayed postovulatory oocyte aging and attenuated the aging-induced defects by increasing the *Sirt1* levels. Thus, it may be worth investigating whether fisetin can improve the quality of oocytes from aged females as well as increasing the success rate of ART procedures by delaying postovulatory oocyte aging. 

## 4. Materials and Methods

### 4.1. Animals

All animal experiments were performed according to the instructions of the Animal Research Institute Committee of South China Agricultural University (approval number: 2021f086). Four-week-old ICR mice were purchased from the Guangdong Medical Laboratory Animal Center. The mice were fed ad libitum and maintained at a constant temperature (25 °C) with 12 h light/dark cycles.

### 4.2. Oocyte Collection and Drug Treatment

MII oocytes were collected from 6–8-week-old female mice, as described previously [17]. Briefly, the mice were injected intraperitoneally with 5–10 IU PMSG, followed by 5–10 IU hCG 46–48 h later to induce oocyte aging. The MII oocytes were retrieved from the ampulla 16 h later and collected in M2 medium (Sigma, St. Louis, MO, USA). The oocytes with surrounding cumulus cells (COCs) were cultured in 500 μL M16 medium (Sigma) in a Nunc™/SPL IVF 4-well dish (ThermoFisher Scientific, Waltham, MA, USA) for 12 h in humid air (37 °C, 5% CO_2_). Fisetin (MedChemExpress, Shanghai, China) was dissolved in DMSO (Sigma) at 80 mM and stored at −80 °C. The optimal fisetin concentration was determined using dose–response gradients. The same volume of DMSO was added into the control groups. The final concentrations did not exceed 1%. 

### 4.3. In Vitro Fertilization

Human tubal fluid (HTF) medium was used for the in vitro fertilization, as described in a previous study [41]. Briefly, HTF medium under mineral oil was pre-cultured for 4 h in humid air. The sperm released from the cauda epididymides were collected in HTF medium for 1 h, and then the sperm were added to the HTF medium contained with COCs at a density of 4 × 10^5^/mL. After 5–6 h, embryos with two pronuclei were identified, and the fertilization rate was calculated. The embryos were cultured in KSOM + AA medium (IVL04, Caisson, Smithfield, UT, USA) under mineral oil in humid air (37 °C, 5% CO_2_), and the 2-cell rate and blastocyst formation rate were calculated after 24 h and 120 h, respectively.

### 4.4. Quantitative Real-Time PCR

Quantitative real-time PCR (qRT-PCR) was performed as previously described [17]. Briefly, 30 oocytes from each sample were lysed in Cell-to-SignalTM Lysis Buffer (Ambion, Austin, TX, USA), and the cDNA was directly generated using a SuperScript^®^ III CellsDirect cDNA Synthesis kit (Thermo Fisher Scientific, Waltham, MA, USA). Quantitative real-time PCR was performed using TB Green^®^ Premix Ex Taq™ (TaKaRa, Beijing, China) on the Applied Biosystems™ QuantStudio™ 5 (Thermo Fisher Scientific). The levels of *Sirt1*, *Sod2*, *Nd2*, *Nd6*, *Co1*, *Co2*, *Atp8*, and *Tfam* transcription were calculated using the 2^−ΔΔCT^ method. The primer sequences are listed in Table S2. Each reaction was repeated in independent triplicates.

### 4.5. Immunofluorescence

Immunofluorescence was performed according to a previous study [41]. Briefly, the samples were fixed in Immunol Staining Fix Solution (Beyotime, Shanghai, China), permeabilized for 5–15 min with Immunostaining Permeabilization Solution with Triton X-100 (Beyotime), and then blocked overnight in QuickBlock™ Blocking Buffer (Beyotime) at 4 °C. The samples were incubated with primary antibodies in QuickBlock™ Primary Antibody Dilution Buffer (Beyotime) for 8 h at 4 °C. After washing the samples three times with PBS-PVA (0.1% PVA diluted in PBS), they were incubated with Alexa Fluor 488 or 555 goat anti-rabbit IgG (H + L) (Invitrogen, Carlsbad, CA, USA) for 2 h at room temperature. After washing the samples three times with PBS-PVA, they were counterstained with DAPI (Beyotime) for 2 min. The primary antibodies used in this study were as follows: rabbit anti-α-tubulin (ab7291, 1:100, Abcam, Cambridge, UK), rabbit anti-SIRT1 (ab189494, 1:100, Abcam), rabbit anti-H3K9me3 (ab176916, 1:1000, Abcam), rabbit anti-H3K56ac (BS74014, 1:100, Bioworld, Louis Park, MN, USA), rabbit anti-H3K4ac (ab176799, 1:500, Abcam), and rabbit anti-Phospho-Histone H2A.X (1:500, #9718, Cell Signaling Technology, New England Biolabs, UK). The samples were covered with glass slides, and images of the samples were captured with a Nikon eclipse Ti-S microscope (Nikon, Tokyo, Japan). The fluorescence intensities were analyzed with ImageJ v1.48 software (National Institute of Health, Bethesda, MD, USA).

### 4.6. Mitochondria Detection

Mitochondria were stained using a MitoTracker™ Red CMXRos Kit (M7512, Thermo Fisher Scientific). The oocytes were incubated for 30 min in MitoTracker™ Red CMXRos diluted in M2 medium (1:1000) away from light. After being fixed in Immunol Staining Fix Solution, the samples were observed under a microscope.

### 4.7. Mitochondrial Membrane Potential Measurement

The mitochondrial membrane potential was measured using a Mitochondrial Membrane Potential Assay Kit with TMRE (C2001S, Beyotime). The oocytes were incubated with TMRE diluted in M2 medium (1:1000) for 30 min at 37 °C away from light. 

### 4.8. Determination of ATP Content

A Bioluminescent Somatic Cell Assay Kit (Sigma) was used to determine the ATP content in the oocytes, as described in previous studies [3,41,57]. Briefly, 20 denuded oocytes from each group were treated according to the manufacturer’s protocol. The standard curves of the ATP contents were plotted using concentrations of 0, 1.0, 2.5, 5.0, 7.5, and 10 pmol. The ATP content for each sample was calculated based on the standard curve.

### 4.9. ROS Measurement

Intracellular ROS in denuded oocytes were measured using a Reactive Oxygen Species Assay Kit (50101ES01, Yeasen, Shanghai, China). The denuded oocytes were incubated in DCFH-DA diluted in M2 medium (1:1000) for 30 min away from light. The samples were observed under a Nikon eclipse Ti-S microscope (Nikon).

### 4.10. GSH Measurement

The GSH level in the oocytes was determined with Cell Tracker Blue CMF2HC (4-chloromethyl-6.8-difluoro-7-hydroxycoumarin, Invitrogen, Carlsbad, CA, USA) according to a previous study [58]. Briefly, the denuded oocytes were incubated with 10 μM cell tracker diluted in M2 medium for 30 min in the dark. After rinsing with PBS, the signals of GSH were measured under a microscope with UV light (370 nm).

### 4.11. Annexin-V Assays

An Annexin V-FITC Apoptosis Detection Kit (C1062S, Beyotime) was used to detect apoptotic cells. The denuded oocytes were incubated with Annexin V-FITC binding solution (195 μL) and Annexin V-FITC (5 μL) for 15 min. After rinsing with PBS, the cells were observed under a microscope (Nikon).

### 4.12. Western Blot

A total of 200 denuded oocytes from each group were pooled together and treated with RIPA buffer (Beyotime, P0013B). After being loaded on 12% acrylamide gels, proteins were transferred to PVDF membranes (Millipore, Bedford, MA, USA) via a wet transfer. The PVDF membranes were cultured in QuickBlock™ Blocking Buffer for Western Blot (Beyotime, P0252) for 30 min at room temperature and then incubated with primary rabbit anti-SIRT1 (ab189494, 1:1000, Abcam) and rabbit anti-GAPDH (AC001, 1:1000, Abclonal, Wuhan, China) overnight at 4 °C. After washing the membranes three times with TBST, they were nurtured with HRP Goat Anti-Rabbit IgG (H+L) (Abclonal) for 2 h at room temperature. To determine the level of SIRT1, the SIRT1 bands normalized to GAPDH were calculated with ImageJ v1.48 software (National Institute of Health, USA).

### 4.13. Statistical Analysis

Data are shown as the mean ± SEM of three independent experiments. One-way ANOVA was used for multiple comparisons, and Student’s *t*-test was used to analyze the differences between two groups. Graphpad Prism 8.0.1 software was used for all statistical analyses. Differences were considered statistically significant if *p* < 0.05.

## 5. Conclusions

Fisetin delayed postovulatory oocyte aging and promoted embryo development after fertilization. Fisetin rescued aging-induced oxidative stress, apoptosis, spindle abnormalities, and mitochondrial dysfunction and restored mitochondrial gene expression by enhancing SIRT1 activity. Our findings provide new insights into the involvement of fisetin, a natural flavonol, in delaying postovulatory oocyte aging. Further research can be conducted to elucidate the specific molecular mechanisms by which fisetin delays postovulatory oocyte aging, explore its efficacy through human studies, evaluate its long-term effects and safety profile, and optimize dosage and delivery methods for potential clinical applications in improving outcomes in assisted reproduction technology procedures.

## Data Availability

The data that support this study are available within the article and from the authors upon request.

## Supplementary Materials

The following supporting information can be downloaded at: https://www.mdpi.com/article/10.3390/molecules28145533/s1, Figure S1. The staining signals of H3K4ac in Fresh, Aged and Fisetin groups. Scale bar. 20 μm. Figure S2. The staining signals of H3K56ac in Fresh, Aged and Fisetin groups. Scale bar. 20 μm. Table S1. The number of staining signal on chromosomes in Fresh, Aged and Fisetin groups. Table S2. Primer sequences for real time PCR.

## Abbreviations

ART	Assisted reproduction technology
COCs	Cumulus–oocyte complexes
DMSO	Dimethyl sulfoxide
DNA	Deoxyribonucleic acid
GAPDH	Glyceraldehyde-3-phosphate dehydrogenase
HTF	Human tubal fluid
h	Hour
hCG	Human chorionic gonadotropin
ICR	Institute of Cancer Research
ICSI	Intracytoplasmic sperm injection
IVF	In vitro fertilization
KSOM+AA	KSOM with amino acid
MII	Metaphase II
mRNA	Messenger RNA
n	Number
PMSG	Pregnant Mare Serum Gonadotropin
qRT-PCR	Quantitative real-time PCR
ROS	Reactive oxygen species
SIRT1	Sirtuin 1
TCA	Tricarboxylic acid
TMRE	Tetramethylrhodamine
%	Percentage
