# Supplementary material for: Fisetin Delays Postovulatory Oocyte Aging by Regulating Oxidative Stress and Mitochondrial Function through Sirt1 Pathway
Source: Molecules. 2023 Jul 20;28(14):5533. doi: 10.3390/molecules28145533 (PMC10384696; doi:10.3390/molecules28145533)
Supplement: Supplementary file 1 [file molecules-28-05533-s001.zip › molecules-2476663-supplementary.pdf]

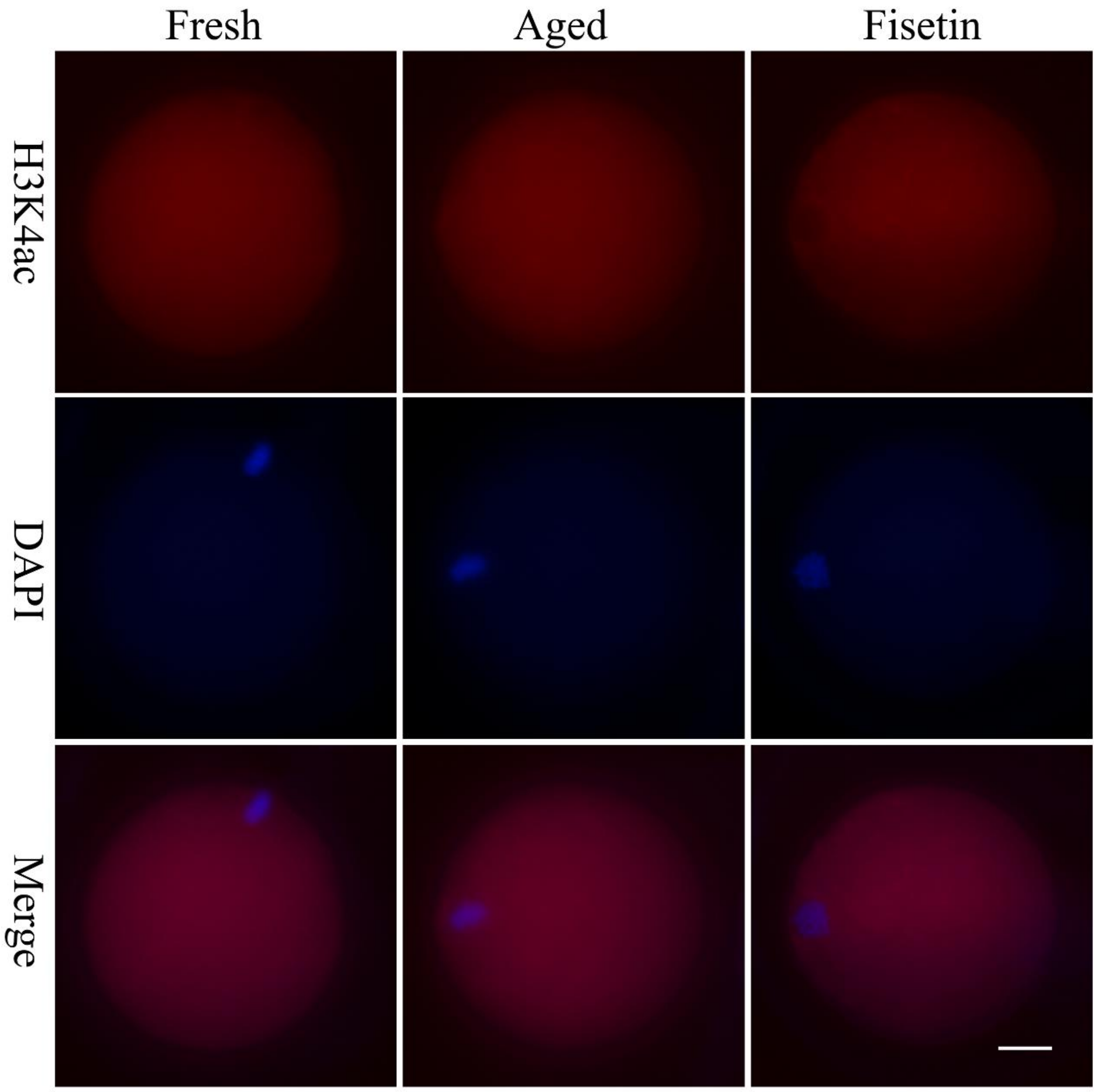

Figure S1. The staining signals of H3K4ac in Fresh, Aged and Fisetin groups. Scale bar. 20 μm.

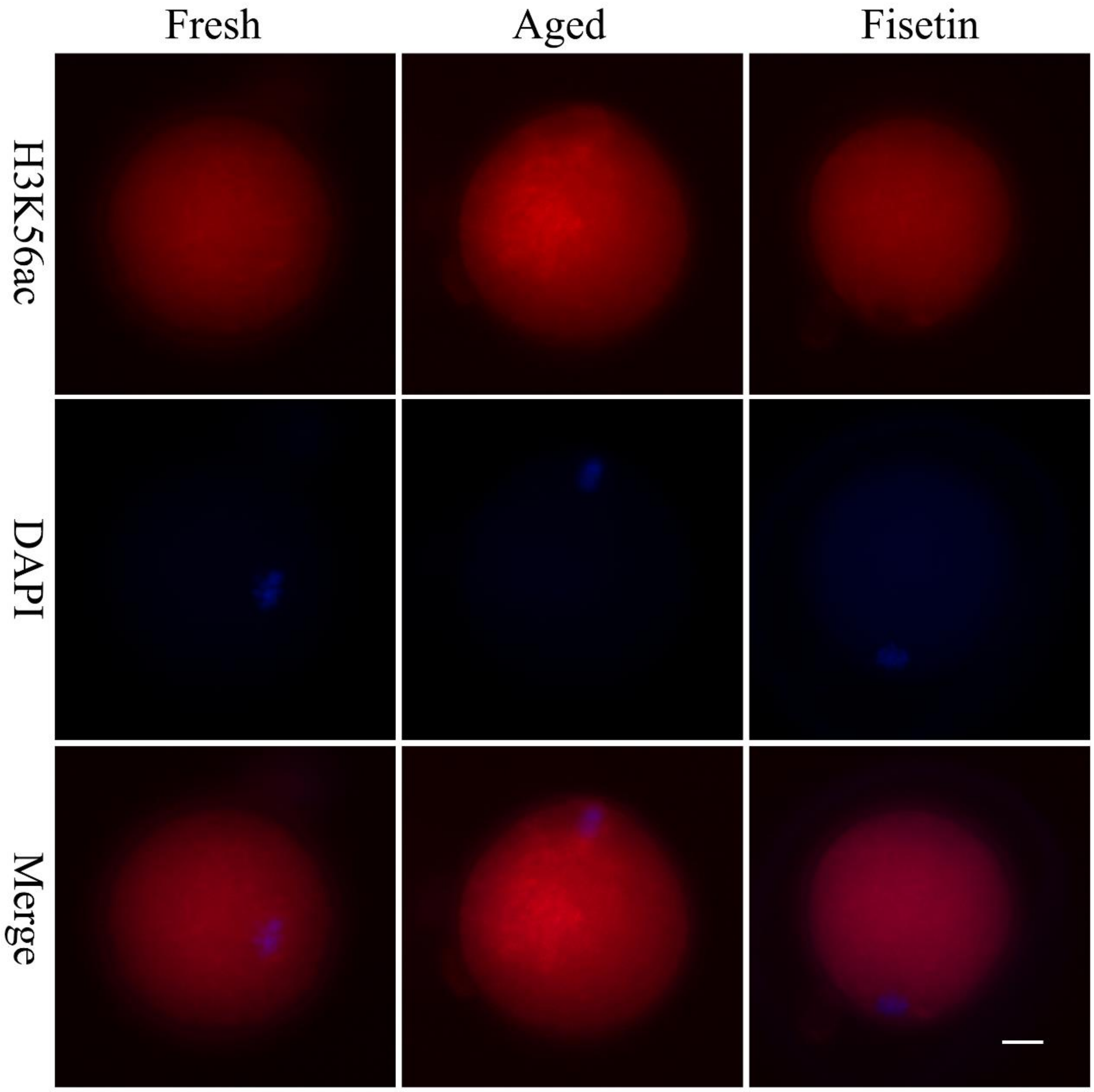

Figure S2. The staining signals of H3K56ac in Fresh, Aged and Fisetin groups. Scale bar. 20 µm.

Table S1. The number of staining signal on chromosomes in Fresh, Aged and Fisetin groups

	Numbers	Fresh	Aged	Aged+Fisetin
H3K4ac	n (total)	30	30	30
	n (signals)	0	0	0
H3K56ac	n (total)	30	30	30
	n (signals)	0	0	0

Table S2 Primer sequences for real time PCR

Gene	Sequence
Gapdh	(F) 5'- TCTTGCTCAGTGTCCTTGC-3' (R) 5'- CTTTGTCAAGCTCATTTCCTGG-3'
Sirt1	(F) 5'- CTCTGAAAGTGAGACCAGTAGC-3' (R) 5'- TGTAGATGAGGCAAAGGTTCC-3'
Sod2	(F) 5'- CAGACCTGCCTTACGACTATGG-3' (R) 5'- CTCGGTGGCGTTGAGATTGTT-3'
Nd2	(F) 5'- TTCGTCACACAAGCAACAGC-3' (R) 5'- GGGGCGAGGCCTAGTTTTAT-3'
Nd6	(F) 5'- CCTCAGTAGCTATAGCAGTCGT -3' (R) 5'- GTTGGTTGTCTTGGGTTAGCA -3'
Co1	(F) 5'- TCGGAGCCCCAGATATAGCA -3' (R) 5'- TTTCCGGCTAGAGGTGGGTA -3'
Co2	(F) 5'- CCTCCACTCATGAGCAGTCC -3' (R) 5'- AACCCTGGTCGGTTTGATGTT -3'
Atp8	(F) 5'- CACAAACATTCCCACTGGCAC -3' (R) 5'- TTGGGGTAATGAATGAGGCAAA -3'
Tfam	(F) 5'- TCTTGGGAAGAGCAGATGGC -3' (R) 5'- TCAGAGATGTCTCCGGATCGT -3'
